# Scaling-Up for the Counter-Rotating Twin Screw Extrusion of Polymers

**DOI:** 10.3390/polym16192720

**Published:** 2024-09-26

**Authors:** Andrzej Nastaj, Krzysztof Wilczyński

**Affiliations:** Polymer Processing Department, Faculty of Mechanical and Industrial Engineering, Warsaw University of Technology, Narbutta 85, 02-524 Warsaw, Poland; andrzej.nastaj@pw.edu.pl

**Keywords:** polymers, extrusion, scale-up, optimization

## Abstract

A novel and original computer scaling-up system for the counter-rotating twin screw extrusion of polymers was developed. In the system, each scaling parameter (scaling criterion) may be considered as an objective function to be minimized for the single parameters or the functional relationships along the length of the screw. Scaling was based on the process simulation, which was performed using the comprehensive (or global) counter-rotating twin screw extrusion program called TSEM (Twin Screw Extrusion Model). The extrusion process was scaled by applying GASES_TWIN_ (Genetic Algorithms Screw Extrusion Scaling) software developed for this purpose using genetic algorithms. Scaling up the extrusion process was carried out to enhance extrusion process throughput according to the scaling criteria specified by the single quantities of polymer melt temperature at the die exit and relative melting length, and by distributions along the screw length of the extrusion parameters of the polymer melt temperature and polymer plasticating. The global objective function had the lowest value for the selected extrusion parameters, which means the minimal differences between the values of the scaled-up processes and extrusion throughput was significantly increased. The solution to the problem of scaling the counter-rotating process presented in this paper complements the existing solutions for optimizing and scaling basic variants of the extrusion process, i.e., flood-fed and starve (metered)-fed single-screw extrusion, as well as co-rotating and counter-rotating twin-screw extrusion.

## 1. Introduction

Computer-aided design and computer-aided engineering systems (CAD/CAE) based on process modeling are very useful in designing polymer processing. However, these systems do not make it possible to optimize the engineering processes according to selected optimization criteria. Extrusion optimization is a very complicated issue because of the very large number of process data (material, geometry, and operation) and optimization criteria (very often contradictory).

Optimization involves generating a multidimensional response space of the output data of the process based on the input data and looking for extreme values in this space (maximal or minimal). Data for optimization may be obtained from experiments or simulation studies, but optimizations with the use of simulation data are much more effective.

There are many optimization methods, e.g., statistical ones. The biggest disadvantage of these methods is the need to study the response space with a very high density of process data and the risk of obtaining local solutions, not global ones.

There are also known methods of modeling and optimizing physical processes based on artificial intelligence techniques (neural networks, genetic algorithms, fuzzy procedures), which provide continuous or discrete solutions obtained as a result of the learning process based on available data. Optimization methods developed using genetic algorithms are of special meaning for the extrusion process.

Genetic algorithms (or evolutionary techniques) are characterized by the following features compared to other optimization methods:

- optimization parameters are not processed directly (they are encoded);
- the search for a solution takes place in a randomly generated population, which allows for avoiding local extrema;
- selection rules are probabilistic in nature;
- a new search surface is specified based on previous experience;
- the objective function is used, not its derivatives.

These methods have been used to develop systems for optimizing the basic variations of the extrusion process, i.e., single-screw extrusion with both flood feed extrusion [1,2,3,4,5,6] and meter feed (starve fed) extrusion [7,8], as well as co-rotating [9,10,11,12] and, more recently, counter-rotating twin-screw extrusion [13].

An important procedure to design physical processes is scaling-up, that is, variation in the scale of the process according to specified criteria, while keeping the scaling data of the target process as close as possible to the data of the reference process. Initially, extrusion scaling was carried out based on single-parameter scaling criteria, which only described selected process characteristics. Scaling up with the use of a mathematical model of the process allows for changing the extrusion scale with the use of the characteristics of the whole process. Optimizing techniques are used here to minimize the differences between the parameters of the scaled processes. Therefore, modeling and optimization solutions are the basis for extrusion scaling.

Extrusion scaling involves defining the geometry and operating conditions of the target machine (extruder) that should reflect the performance of the reference machine (extruder). Scaling rules enable the design of large extruders using the results of laboratory-scale studies.

A number of different procedures have been developed to scale up the extrusion process and are presented in several monographs, e.g., [14,15,16,17,18,19,20] and in a number of papers.

The analytical models of the extrusion process were mostly used to correlate the large (target) and small (reference) primary scaling parameters (the screw rotational speed and basic screw dimensions and the diameter, channel depth, and length, respectively) in terms of an exponent of the ratio of the screw diameters of the reference and target extruder

(1) $$\frac{Y_{target}}{Y_{ref}}={\left(\frac{D_{target}}{D_{ref}}\right)}^{sf}$$

where *Y*_ref_ and *Y*_target_ are the reference and target scaling parameters, respectively, *D*_target_ and *D*_tref_ are the reference and target screw diameters, and *sf* is the scale-up parameter.

Carley and McKelvey [21] were the first to carry out scaling for extrusion. They considered the melt transport section of a screw and developed scaling formulas for screws in which the screw channel dimensions were increased proportionally to the screw diameter ratio while keeping a constant screw speed.

Furthermore, a few other scaling rules have been proposed for single-screw extrusion [22,23,24,25,26,27,28,29,30,31,32]. Pearson [25] was the first to perform a complete analysis of extrusion scaling, taking into account solid transport, melting, and melt transport, and acceptable scaling was obtained if the Graetz number, Brinkman number, and Nahme number were kept constant in the screw functional sections.

Covas and Cunha [33] analyzed existing scaling rules and concluded that the current rules:

- may cover only single scaling criteria (for example, the polymer melting rate or polymer pumping rate) and single steps of the extrusion process (for example, melting or melt transport);
- may consider only a few geometrical or operational data, e.g., the screw speed or screw dimensions;
- are based on the very simplified models of the process.

Therefore, more effective scale-up procedures are desired that are developed using much more accurate mathematical descriptions of the process and enable

- taking into account several scaling criteria simultaneously;
- the selection of single quantities (for example, the melting rate) or functions (for example, polymer solid bed distribution over the screw) as scaling criteria.

Covas and Cunha stated [33] that these goals may be obtained by treating extrusion scaling as a multi-objective optimizing problem where the goal is to determine the geometry/operational data of the target machine in such a way that the performance data of both machines are as close as possible. The purpose of scaling is to minimize the difference between the selected response data of the target and reference machines. The geometry/operational data of the reference machine are established, and those of the target machine are sought.

Implementing these new scaling concepts requires the following actions:

- simulating an extrusion to obtain the response parameters of a reference machine with a given set of process data (input data ≥ modeling ≥ results);
- indication of scaling-up criteria (results ≥ scaling criteria);
- determining the fixed parameters of the target machine; for example, the screw diameter *D_screw_* and the ratio of the screw length to the screw diameter *L_screw_*/*D_screw_*;
- carrying out the scaling-up procedure by minimizing the differences between the specified data of the target machine and reference machine (optimization ≥ geometry/operational parameters).

**Figure 1:**
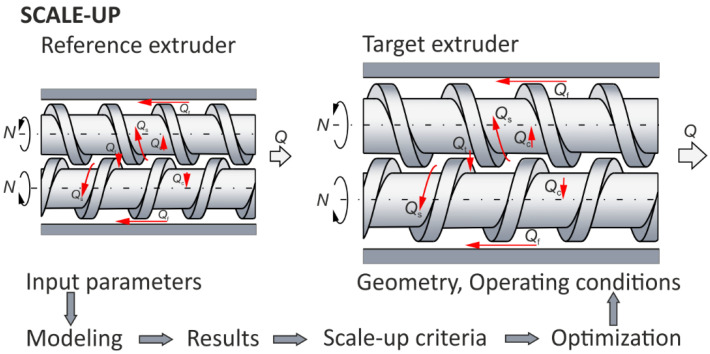
The optimizing/modeling idea of scaling for extrusion, e.g., counter-rotating: N—screw rotational speed, Q—extrusion throughput, and leakage flows, Q_c_—calendering flow, Q_f_—flight flow, Q_t_—pressure (tetrahedral) flow, Q_s_—side (lateral) flow (adopted with permission from: Wilczyński, K. *Rheology in Polymer Processing. Modeling and Simulation*; Carl Hanser Verlag: Munich 2021 [34]).

In summary, this method involves extrusion modeling/simulation, the definition of scaling-up criteria, and multi-objective optimizing. The scaling-up criteria are calculated for the target machine and compared with equivalent criteria for the reference machine, and the quality of each calculation is assessed. The search for a solution is iteratively repeated until a satisfactory minimization of the differences is achieved. This scaling idea is presented in Figure 1.

The key to good process scaling is the proper selection of scaling criteria, which are quantities characterizing the process, in relation to which we make a comparison of the target process to the reference process. The differences in these quantities for the target process and the reference process should be minimal. Typical scaling criteria are the average shear rate, the average shear stress, the polymer solids transport rate, the polymer plasticating rate, the polymer pumping rate, residence time distribution, and energy consumption, as proposed by Rauwendaal [30] and Potente [31] as well as by Covas and Cunha [33].

Most scaling criteria are defined by single parameters, but sometimes it may make sense to take into account their distributions along the screw. An example is the solid bed distribution, i.e., the solid bed profile (*SBP*) defined by the ratio of the bed width to the channel width.

Each of the scaling criteria can be treated as an objective function *F_i_*, which should be minimized, respectively, for single values or functional relationships (axial profiles), that is [33]:

(2) $$F_{i}=\frac{\left|Y_{i}-Y_{i}^{r}\right|}{Y_{i}^{r}}$$

(3) $$F_{i}=\frac{\sum_{k-1}^{K}\frac{\left|Y_{i,k}-Y_{i,k}^{r}\right|}{Y_{i,k}^{r}}}{K}$$

where *F_i_* is the fitness of the *i*-criterion, $Y_{i}$ and $Y_{i}^{r}$ are the single values of the *i*-criterion for the target/reference extruders, and $Y_{i,k}$ and $Y_{i,k}^{r}$ are the values of the *i*-criterion on the *k*-location (along the screw length) for the target/reference extruders.

Using this multi-objective optimization approach [1,2,3,4,9,10,11,12], Covas and Cunha [33] performed the full scale-up of single-screw extrusion in terms of both screw geometry and operating conditions. This approach makes it possible for different criteria to be considered at the same time and their relative relations as well. Multi-criteria scaling is more effective than scaling using a single process response because the optimizing algorithm finds results that satisfy multiple criteria simultaneously.

The scaling of extrusion using modeling/simulation has so far been limited to classical flood-fed single-screw extrusion and co-rotating twin-screw extrusion [33,35,36,37,38,39,40]. This scaling was based on the extrusion models [3,41,42,43] for single-screw extrusion and the models [44,45,46,47,48,49] for co-rotating twin-screw extrusion. The state-of-the-art in this field has been discussed in the review paper [50]. Recently, the first approach by which to solve this problem for starve (metered)-fed single-screw extrusion was discussed by the authors [51]. This scaling was based on the extrusion models [52,53,54,55]. The counter-rotating twin-screw process has not yet been scaled up.

In this paper, an original computer scaling-up method for counter-rotating twin screw extrusion is presented. The scale-up criteria were considered as an objective function *F*_i_ to be minimized for the single values (Equation (2)) and for the functional relationships along the screw (Equation (3)). Scaling-up was carried out by process simulation with the use of the extrusion mathematical model TSEM (Twin Screw Extrusion Model) [34]. The process was scaled up by using novel GASES_TWIN_ (Genetic Algorithms Screw Extrusion Scaling) procedures that used genetic algorithms. Examples of scaling-up were presented to enhance the extrusion throughput according to the scale-up criteria determined by the single quantities of polymer temperature at the die outlet and relative plasticating (melting) length and by the process parameter distributions of the polymer melt temperature and polymer plasticating. To the best of our knowledge, it is the first system of scaling the counter-rotating twin screw extrusion that uses process simulations.

## 2. Counter-Rotating Twin-Screw Extrusion

Twin (double)-screw extrusion may be co-rotating (the screws rotate in the same direction) or counter-rotating (or contrary-rotating) extrusion (the screws rotate in the opposite direction).

**Figure 2:**
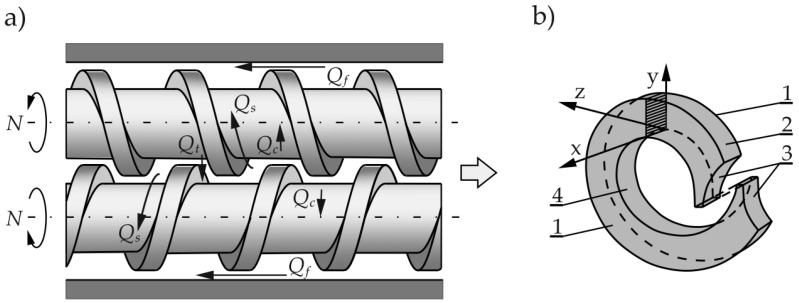
Scheme of the counter-rotating twin-screw extrusion: (**a**) leakage flows, Q_c_—calendering flow, Q_f_—flight flow, Q_t_—pressure flow, Q_s_—side (lateral) flow, (**b**) C-shaped chamber, 1—side of the screw flight, 2—barrel, 3—front of the screw flight, 4—screw root (adopted with permission from: Wilczyński, K. *Rheology in Polymer Processing. Modeling and Simulation*; Carl Hanser Verlag: Munich 2021 [34]).

Counter-rotating twin-screw machines are primarily used to process thermally sensitive polymers such as polyvinyl chloride (PVC) and, compared to other variants of extrusion, provide better feeding of the machine with powdered or slippery material. Co-rotating twin-screw machines have specialized applications, such as compounding, filling, or reinforcing polymeric materials. A scheme of a counter-rotating twin screw extruder is shown in Figure 2.

Counter-rotating extruders are usually meter (or starve)-fed. Transport of the material in these is different from that in single-screw and co-rotating twin screw machines. This transport is due to a positive displacement mechanism that does not occur in other machines. The material in the counter-rotating extruders is moved in a C-shaped chamber (Figure 2b), and there are also some leakage flows observed (Figure 2a), that is, calendering flow Q_c_, flight flow Q_f_, pressure flow Q_t_, and side (lateral) flow Q_s_.

Some fundamental monographs presented the state-of-the-art on the modeling of extrusion, e.g., [14,56,57,58,59,60], as well as several papers, e.g., [46,61,62,63,64]. Wilczyński et al. discussed these in review papers [65,66].

The basics of counter-rotating twin-screw extrusion were first discussed many years ago [67,68,69,70], and several designs of these machines were later developed [71,72]. These machines were first described by Kiesskalt [68] and Schenkel [71] as positive displacement pumps. Doboczky [73] and Janssen [74] considered the leakage flows. White and Adewale [75] modeled the polymer transport, including the intermeshing level of the screws. FEM (Finite Element Method) simulations were first presented by Li and Manas-Zloczower [76] and by Kajiwara et al. [77]. Hong and White [78,79] performed a FAN analysis (Flow Analysis Network) for non-Newtonian flow and introduced the concept of screw characteristics, which made it possible to model the flow for different screw designs and the calculation of pressure, fill factor, and temperature distributions. Wilczyński and Lewandowski [80] performed fully 3D non-Newtonian FEM modeling to develop the screw pumping characteristics, which can be implemented into the global model of the process.

Studies on the transport and plasticating of polymers in counter-rotating machines have been very limited [73,74]. Wilczyński and White first experimentally investigated and modeled the plasticating process [81,82]. Further studies were performed by Wang and Min [83,84] and Wilczyński et al. [85].

White et al. [78,79,81,82] developed a theory that allowed for the prediction of both the polymer pumping rate and the polymer solid distribution in this process. The first global models were presented by Wilczynski et al. [86,87,88] to predict solids conveying, plasticating, and melt conveying. Jiang et al. [89] built a model for counter-rotating twin-screw extrusion with flood feeding.

Lewandowski et al. [90] developed a model for the whole counter-rotating twin screw extruder with the die. This conception is based on the combination of melt transport models with plasticating and solid transport models. 3D non-Newtonian FEM calculations for melt transport were carried out to develop the screw pumping characteristics, which were used for the global model. To our knowledge, this was the first and is so far the only available global model of a counter-rotating twin screw extruder with the use of 3D non-Newtonian FEM calculations. This model has been improved and extended and is a part of the computer system named the Multi Screw System [34].

The computer model of counter-rotating twin-screw extrusion TSEM that was used in this study was validated experimentally using the broad range of material data, extrusion process operating data, and screw geometry (also for pressure development), e.g., [81,82,85,86,88,90,91], and among these studies, the reference screw and polypropylene (PP).

**Figure 3:**
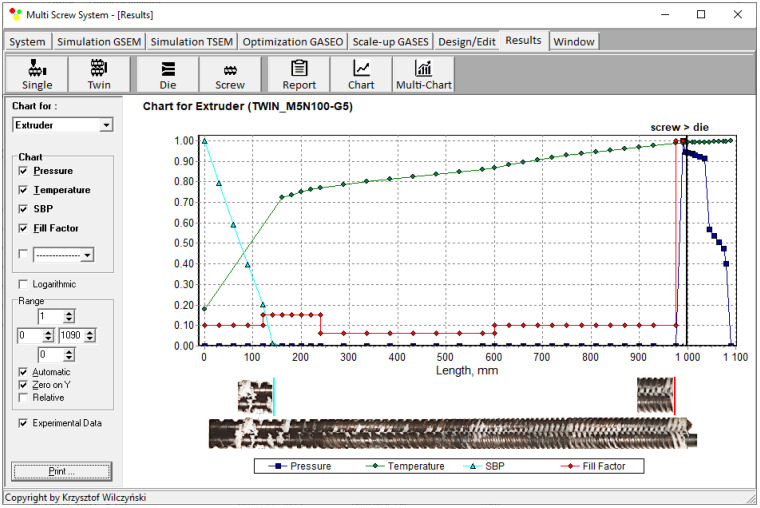
Extrusion process characteristics, simulation using the TSEM program—twin screw extrusion, Q = 5 kg/h, screw speed N = 100 rpm: Pressure (dark blue color line), Temperature (green color line), Solid Bed Profile (SBP, blue color line), Fill Factor (red color line).

**Figure 4:**
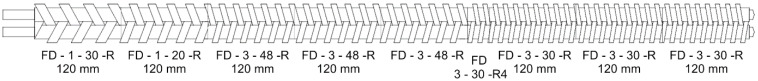
Counter-rotating twin screw configuration: FD—thick flighted element.

An example of the calculation using TSEM software is depicted in Figure 3 for the screw system illustrated in Figure 4. The experiment was carried out for an extrusion of polypropylene (PP) at a screw speed of N = 100 rpm and an extrusion throughput of G = 5 kg/h. The dimensionless process characteristics are shown by pressure (P—dark blue color) distribution, temperature (T—green color) distribution, polymer plasticating distribution, shown by the Solid Bed Profile (SBP—blue color), and screw filling distribution shown by the Fill Factor (FF—red color). It can be seen that pressure is built up only in the area with completely filled screws. However, this pressure is high enough to push the material through the die. The screws are only completely filled with a polymer in the short, last section before the die. In the rest of the machine, the screws are filled partially, only by 10–15%. The degree to which the screw is filled with material results from the screw geometry, screw speed, and feed rate.

Counter-rotating twin-screw extrusion is usually performed with metered feeding (starve-fed extrusion). In this case, the extrusion output is settled by the operators. For a fixed extruder feed rate, increasing the screw rotation speed causes the degree of filling the screw with polymer to decrease, but the extrusion throughput does not change. At a fixed screw rotation speed, changing the extruder feed rate causes the extrusion throughput to change and the degree of filling the screw with polymer to change too.

## 3. Scaling-Up Procedure

The genetic algorithm method belongs to the group of probabilistic optimization methods. The main advantage of this method is the low probability of getting stuck in a suboptimal solution, and at the same time, its effectiveness does not depend on the starting point of the search. The most important disadvantage of genetic algorithms is that you cannot be sure whether the optimal solution has been found.

A literature analysis does not clearly indicate the best form of implementing genetic algorithm procedures (selection, crossover, mutation, etc.). Our experience shows that tournament selection is one of the best methods, as it allows. for finding the optimal solution after reviewing a relatively small number of response spaces (solutions).

**Figure 5:**
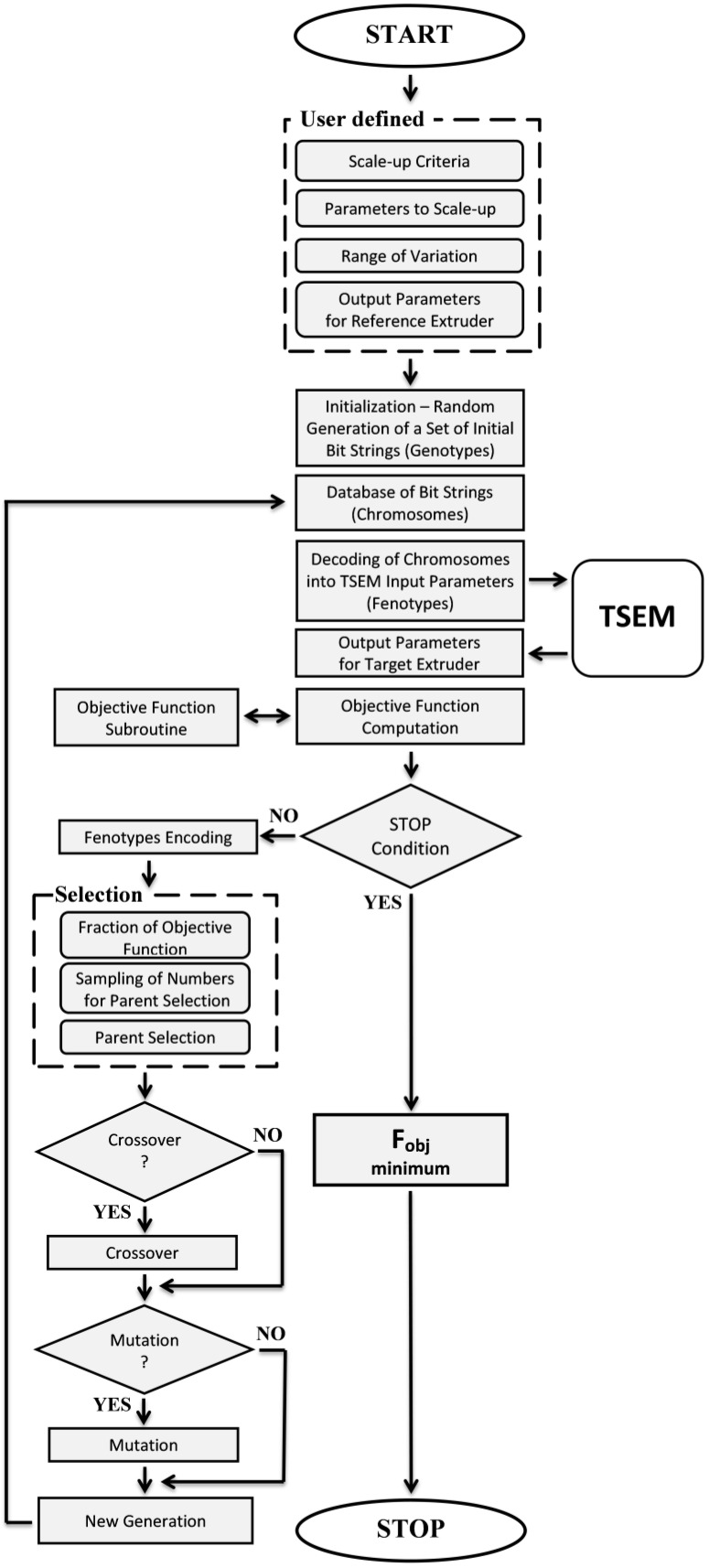
Flowchart of the GASES_TWIN_ program algorithm (in collaboration with the TSEM program).

In this research, a scaling-up system, GASES_TWIN_ (Genetic Algorithms Screw Extrusion Scaling), using genetic algorithms, was developed. The source of data for scaling up were simulations made by the TWIN (Twin Screw Extrusion Model) system. A flowchart of the GASES_TWIN_ program algorithm (in collaboration with the TSEM program) is shown in Figure 5.

The scaling procedure begins with the user establishing the scaling criteria, the process parameters to be scaled, the range of changes to these parameters, and the output parameters of the reference extruder.

The GASES_TWIN_ scaling-up program, in cooperation with the TSEM simulation program, makes it possible to scale up a twin-screw extrusion with a varied amount of process variables, with various scale-up criteria specified by the single process data as well as by the process data distributions. The precision of investigating the response surface is defined by the number of divisions of parameter ranges, which result from the length of writing these numbers in a binary system. In GASES_TWIN_, the length of the binary series is adjustable, and its maximum length is 255 characters. This allows the range of each parameter to be divided into 2^255^ values.

We would like to point out that the algorithm used in the work is not limited to a specific number of iterations. The algorithm ends after the same data set with the smallest objective function value occurs 100 times. With such a defined stopping condition, the probability of finding the optimal solution is very high.

A Tournament Selection is used as the method of selection. This method is a technique used in genetic algorithms to choose individuals from a population. It works by holding mini-competitions, called tournaments, among a small group of randomly selected individuals (chromosomes). The winner of each tournament, the one with the highest fitness, is selected for crossover. Selection pressure, which refers to the likelihood of an individual being chosen for a tournament, can be easily adjusted through the tournament size. Larger tournaments increase the chance of strong individuals being selected because there is a higher probability of them being present in each competition. This effectively reduces the chances of weaker individuals being chosen.

The entire Tournament Selection procedure can be summarized in the following steps:

1. Define Tournament Size: specify the number of individuals that will compete in each mini-competition (tournament).
2. Determine Selection Count: set the total number of individuals you need to select for the next generation.
3. Random Subset Selection: randomly choose a group of individuals from the population; each individual has an equal chance of being selected.
4. Fitness Evaluation: evaluate the fitness of each individual within this randomly chosen subset.
5. Winner Selection: identify the individual with the highest fitness score in the subset; this individual becomes a parent for the next generation.
6. Repeat: repeat steps 3–5 until the desired number of individuals (as defined in step 2) have been chosen for the next generation.

This method offers several advantages over other genetic algorithm selection methods, such as fitness proportionate selection and reward-based selection. These advantages include efficient coding, compatibility with parallel architectures, and adjustable selection pressure.

**Figure 6:**
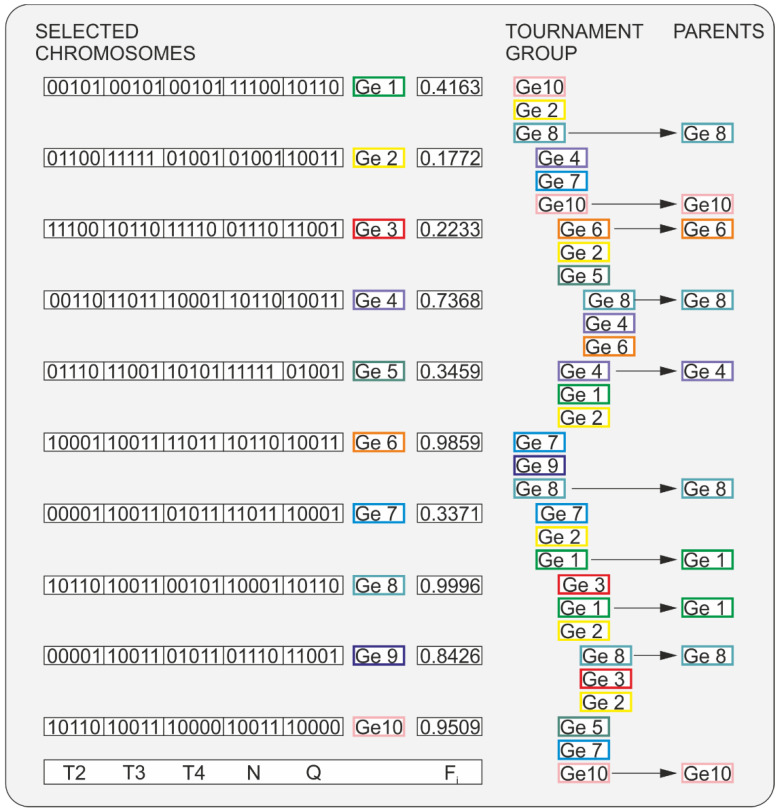
Selection of initial population and evaluation of chromosome adaptation: T2, T3, T4—barrel temperature in the section II, III, IV; N—screw speed; Q—feeding rate (extrusion throughput), F—objective function.

An operation scheme for Tournament Selection is depicted in Figure 6. For each genotype, the objective function value is calculated. Genotype Ge2 has the lowest objective function value, F_Ge2_ = 0.1772, while genotype Ge8 has the highest objective function value, F_Ge8_ = 0.9996. Then, a tournament mini-group of three genotypes is randomly chosen (first tournament). From this group, the genotype with the highest objective function value is selected. In the presented example, genotype Ge8 has the highest objective function value, F_Ge8_ = 0.9996, which therefore proceeds to the next step. Then, a tournament mini-group of three genotypes is randomly chosen again (second tournament). In the chosen group, genotype Ge10 has the highest objective function value, F_Ge10_ = 0.9509, which proceeds to the next step. The selection procedure is repeated until ten genotypes are obtained, which will form the new parent group. In the given example, these will be the genotypes: Ge8, Ge10, Ge6, Ge8, Ge4, Ge8, Ge1, Ge1, Ge8, Ge10. They will then be subjected to crossover and mutation to create a new set of genotypes. The whole process is repeated until the termination condition is met (no improvement in the value of the objective function for 100 iterations).

**Figure 7:**
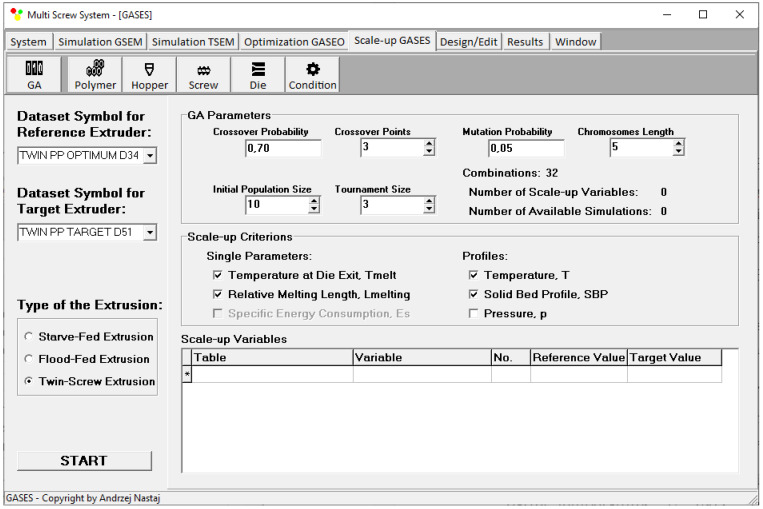
Input data of the scaling-up system.

Scaling up is defined by genetic algorithm parameters, i.e., the number of scaling variables, the starting population size, the tournament size, the chromosome length, the crossover probability, crossover points, and mutation probability. An effect of the GA parameters on the results has not been investigated here. However, it was observed that this effect is not overly important. Moreover, it was observed that GA parameters have a significant impact on the computation time. In this paper, these parameters were established based on the literature [92,93,94,95] and our experiences [13,51]. The different weights of the criteria are not available here. This issue is depicted in Figure 7.

## 4. Scaling Up

### 4.1. Research Program

**Figure 8:**
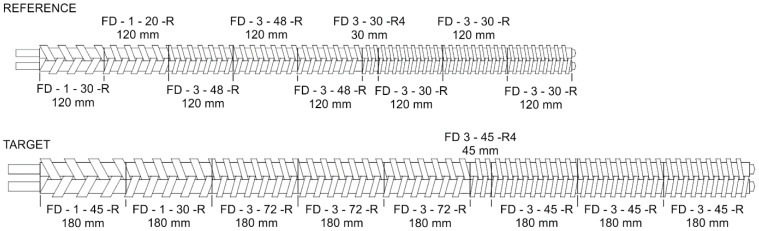
Reference and target screw configurations: FD—thick flighted element.

Scaling up was carried out for counter-rotating twin-screw extrusion to enhance the process throughput according to the scale-up criteria specified by the single variables of polymer temperature at the die exit and the relative melting length, and by distributions along the screw length of the extrusion variables of the polymer melt temperature and polymer melting. The relative melting length is defined by the ratio of the screw length required for melting to the screw length. The investigation program included a scale-up of the extrusion from the level of the reference extruder (Leistritz LSM 30/34) with the screws with diameters of D_ref_ = 34 mm and the distance between the center lines B_ref_ = 30 mm to a level of the target extruder with screws with diameters of D_target_ = 51 mm and the distance between the center lines B_target_ = 45 mm, while keeping the ratio of the screw length/diameter (L/D) constant. The screw geometries of the reference and target extruders are shown in Figure 8 and Table 1.

Polypropylene (PP) was used in the study, as in other studies on the optimization of counter-rotating extrusion [13]. This material has a solid density of *ρ_s_* = 0.904 g/cm^3^ and a mass flow rate of MFR = 2.7 g/10 min (230 °C, 2.16 kg). The material rheological data were obtained with the use of capillary rheometry and were described with the use of the Klein model, that is

(4) $$ln\eta =a_{0}+a_{1}ln\dot{\gamma }+a_{11}{ln}^{2}\dot{\gamma }+a_{12}T{ln}^{2}\dot{\gamma }+a_{2}T+a_{22}T^{2}$$

where *η* means the viscosity, $\dot{\gamma }$ means the shear rate, *T* means the temperature, and *a*_0_, *a*_1_, *a*_11_, *a*_12_, *a*_2_, *a*_22_ are the parameters of the model (*a*_0_ = 14.0587, *a*_1_ = −0.4535, *a*_11_ = −0.0281, *a*_12_ = 2.71 × 10^−4^, *a*_2_ = −0.0316, *a*_22_ = 4.45 × 10^−5^).

### 4.2. Scale-Up of Counter-Rotating Twin-Screw Extrusion

The scaling was carried out with respect to the extrusion process, the operating variables of which were obtained by optimization. These (optimized) variables were the screw speed and barrel temperatures. The optimization was made in order to maximize extrusion throughput *Q*ma_max_ (kg/h), minimize the polymer melt temperature at the die exit *T*_out_ (°C), and minimize the polymer plasticating length *L*_pl_ (mm) within the scope of the screw speed, *N* = 40 ÷ 240 rpm, the barrel temperature in the subsequent extruder sections, *T*_I_ = 180 °C, *T*_II_ = 180 ÷ 240 °C, *T*_III_ = 180 ÷ 240 °C, *T*_IV_ = 180 ÷ 240 °C, and the feed rate, *Q* = 1 ÷ 100 kg/h. The following values of the optimization criteria weights were assumed: *w*_Q_ = 0.5, *w*_Tout_ = 0.1, *w*_Lpl_ = 0.4.

The global objective function was used in the form

(5) $$F_{i}=w_{Q}\cdot Q_{{i\_}_{norm}}+w_{Tout}\cdot T_{outi\_norm}+w_{Lpl}\cdot L_{pli\_norm}$$

where the output variables (optimization criteria) were normalized as

(6) $$Q_{i\_norm}=\frac{Q_{i}-Q_{min}}{Q_{max}-Q_{min}}$$

(7) $$T_{outi\_norm}=\frac{T_{outmax}-T_{outi}}{T_{outmax}-T_{outmin}}$$

(8) $$L_{pli\_norm}=\frac{L_{pl\;max}-L_{pli}}{L_{plmax}-L_{plmin}}$$

where *F*_i_ means the global objective function, *Q*_i_norm_ means the normalized flow rate (extrusion throughput), *w*_Q_ means the weight of flow rate, *T*_out i_norm_ means the normalized polymer melt temperature at the die exit, *w*_Tout_ means the weight of the polymer melt temperature at the die exit, *L*_pl i_norm_ means the normalized polymer melting length, *w*_Lpl_ means the weight of the polymer melting length, and *i* means the number of the next value from the data set.

The highest value of objective function was obtained at a screw speed of *N* = 240 rpm and barrel temperatures of *T*_I_ = 180 °C, *T*_II_ = 180 °C, *T*_III_ = 187.5 °C, and *T*_IV_ = 195 °C. These optimal data, according to the adopted criteria of maximum output, the minimum polymer melt temperature at the die exit, and the minimum polymer melting length, correspond to the output data of the process, i.e., mass flow rate *Q* = 21.88 kg/h, polymer melt temperature *T*_melt_ = 207 °C, and relative melting length *L*_melting_ = 0.242, that is, the ratio of the screw length needed to melt the polymer to the screw length.

**Figure 9:**
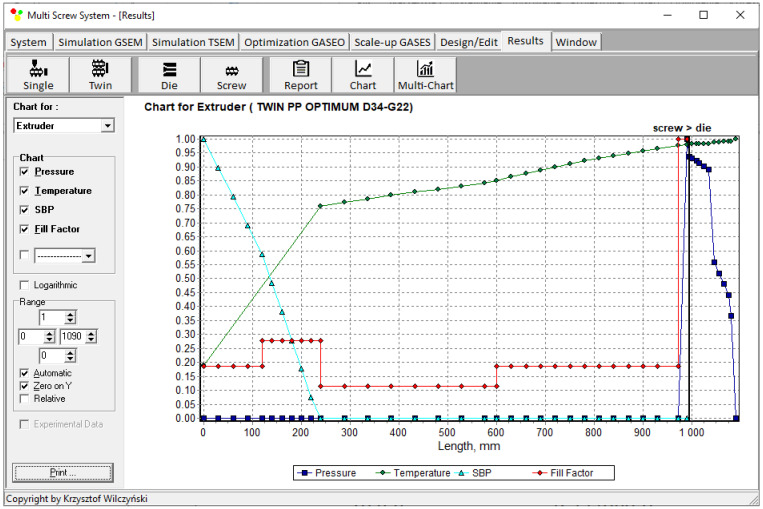
Simulation of counter-rotating extrusion (TSEM): general process characteristics for the reference machine at optimal process parameters: Pressure (dark blue color line), Temperature (green color line), Solid Bed Profile (SBP, blue color line), Fill Factor (red color line).

**Figure 10:**
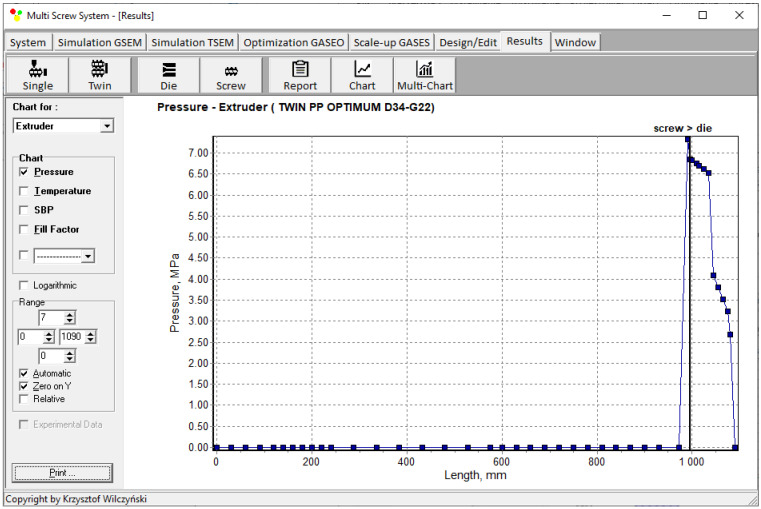
Simulation of counter-rotating extrusion (TSEM): pressure profile for the reference machine at optimal process parameters.

**Figure 11:**
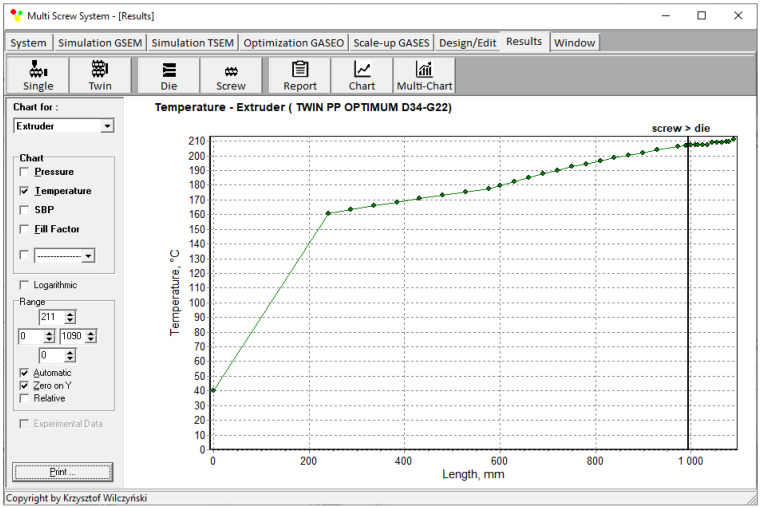
Simulation of counter-rotating extrusion (TSEM): temperature profile for the reference machine at optimal process parameters.

**Figure 12:**
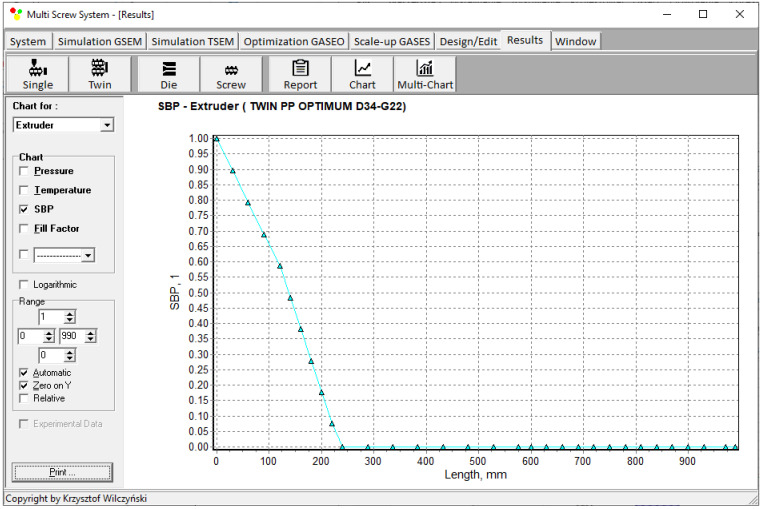
Simulation of counter-rotating extrusion (TSEM): Solid Bed Profile (SBP) for the reference machine at optimal process parameters.

**Figure 13:**
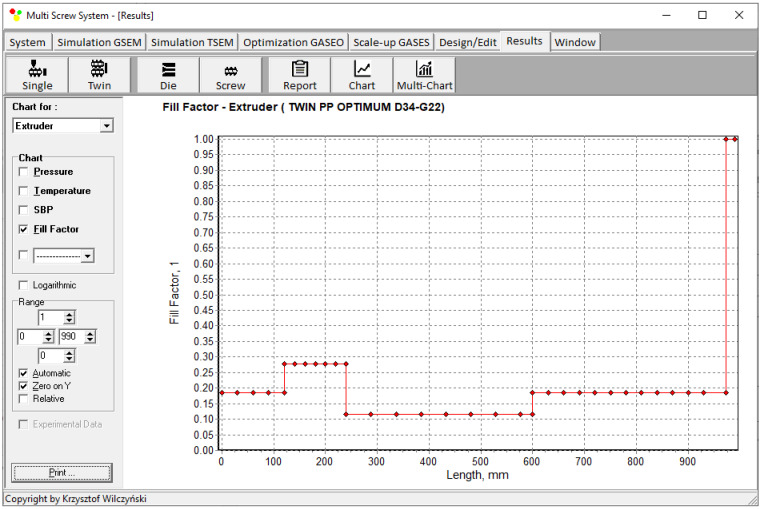
Simulation of counter-rotating extrusion (TSEM): screw filling profile (Fill Factor, FF) for the reference machine at optimal process parameters.

Simulations for the reference machine at optimal operating data are shown in Figure 9 in the form of general process characteristics (dimensionless), which include pressure/temperature distributions, the melting profile (Solid Bed Profile, *SBP*), and the screw fill profile (Fill Factor, *FF*). A fill factor *FF* = 1 indicates that the channel is filled up, *FF* = 0 means that the channel is empty, and 0 < *FF* < 1 indicates that the screw is partially filled. Individual process characteristics of pressure, temperature, and polymer melting screw filling are depicted in Figure 10, Figure 11, Figure 12 and Figure 13. It can be seen that pressure is developed only in the area of completely filled screws. However, this pressure is high enough to push the material through the die. It is also interesting that the screws are completely filled with polymer only in the short, final section before the die. In the rest of the extruder, the screws are filled by only 10–30%. The degree to which the screw is filled with material results from the screw geometry, screw speed, and feed rate.

With respect to such an optimized reference process, the scaling was performed in the same input data scope of the screw speed, N = 40 ÷ 240 rpm, and the barrel temperatures in the subsequent extruder sections: T_I_ = 180 °C, T_II_ = 180 ÷ 240 °C, T_III_ = 180 ÷ 240 °C, T_IV_ = 180 ÷ 240 °C, while modifying the feed rate range to Q = 50 ÷ 200 kg/h.

The scaling was performed according to the single parameter criteria of the polymer temperature *T_melt_* and the relative “melting length” *L_melting_*, i.e., the ratio of the screw length needed to melt the polymer to the screw length, and according to the functional criteria of the polymer melt temperature profile and the melting profile, that is, the solid bed profile (*SBP*).

A global objective function was formulated as

(9) $$F_{i\;s}=\left|1-\frac{T_{{melt}_{A}}}{T_{{melt\;B}_{i}}}\right|+\left|1-\frac{L_{melting\;A}}{L_{melting\;B_{i}}}\right|+\frac{\sum_{k=1}^{n}\left|1-\frac{T_{A\;k}}{T_{B\;ik}}\right|}{n}+\frac{\sum_{k=1}^{n}\left|1-\frac{{SBP}_{A\;k}}{{SBP}_{B\;ik}}\right|}{n}$$

where *F_i_*_s_ means the global objective function for scaling; *T*_melt A_, *T*_melt B *i*_ are the polymer melt temperatures at the die exit for the reference and target machine, respectively; *L*_melting A_, *L*_melting B *i*_ are the relative melting lengths for the reference and target machine, respectively; *T*_A_, *T*_B *i*_ are the polymer melt temperature in the reference and target machine, respectively; *SBP*_A_, *SBP*_B *i*_ are the polymer melting profile for the reference and target machine, respectively; *i* is the number of the next value from the data set, *n* is the number of the next value in the profile.

**Figure 14:**
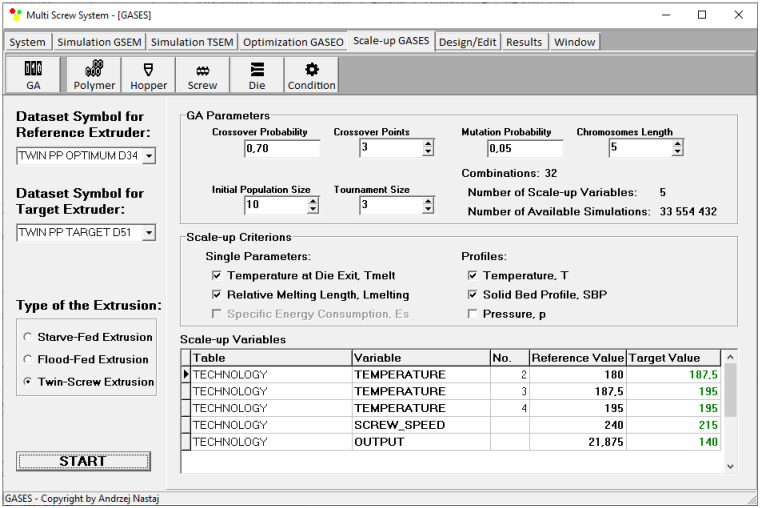
Scaling-up results for counter-rotating extrusion: TEMPERATURE—barrel temperature in the subsequent extruder sections.

**Figure 15:**
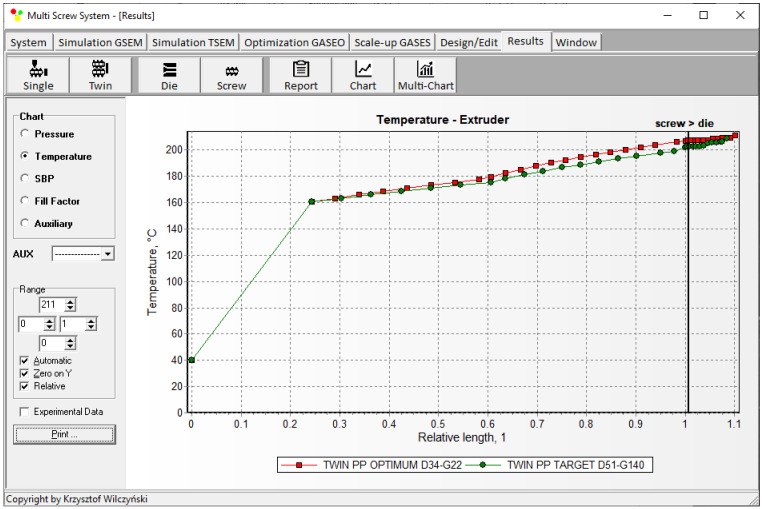
Counter-rotating twin screw extrusion: temperature distribution for the reference (red color) and target (green color) machine.

**Figure 16:**
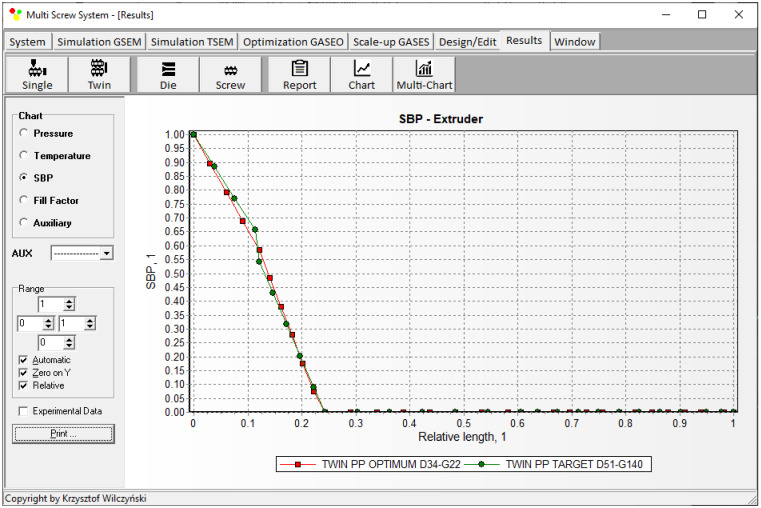
Counter-rotating twin screw extrusion: solid bed distribution (SBP) for the reference (red color) and target (green color) machine.

**Figure 17:**
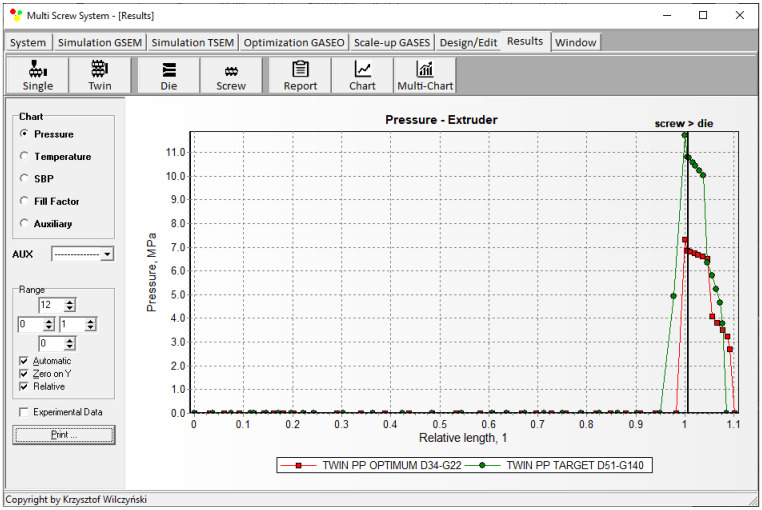
Counter-rotating twin screw extrusion: pressure distribution for the reference (red color) and target (green color) machine.

**Figure 18:**
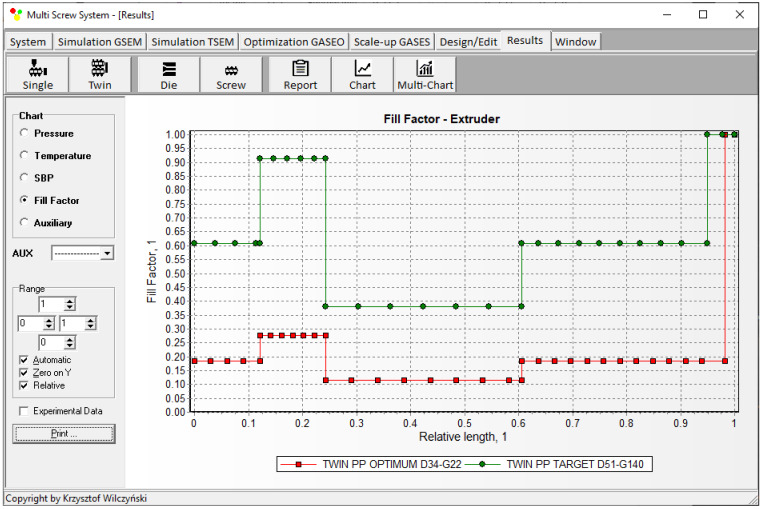
Counter-rotating twin screw extrusion: fill factor distribution for the reference (red color) and target (green color) machine.

The scaling results are presented in Table 2 and Figure 14, Figure 15, Figure 16, Figure 17 and Figure 18. The lowest value of the objective function (Equation (9)), that is, the minimum difference between the reference and target process parameters, was obtained at a screw speed of *N* = 215 rpm for the barrel temperatures: *T*_I_ = 180 °C, *T*_II_ = 187.5 °C, *T*_III_ = 195 °C, *T*_IV_ = 195 °C, at a feed rate of *Q* = 140 kg/h. These parameters correspond to the output variables of the process, i.e., the polymer melt temperature T_melt_ = 202 °C and the relative melting length L_melting_ = 0.242. The differences between the variables of the reference process and the target process are small (Table 2). It can therefore be stated that these processes are similar in terms of the specified criteria. The temperature and melting profiles are also similar, which are presented in Table 2 and Figure 15 and Figure 16. By scaling up, a significant increase in extrusion throughput was obtained, which means that the scaling goal was achieved. In the target process, the pressure and the degree of screw filling also increased (Figure 17 and Figure 18). The reason for that is an increase in extrusion throughput. When extrusion throughput (polymer flow rate in the machine) increased, the filling of the screw also increased if the screw speed was kept constant. Similarly, the pressure increased in the plasticating unit (in the screw and in the die) since more polymer was pushed through the die in the unit time.

## 5. Conclusions

An original computer scaling-up system for counter-rotating twin-screw extrusion was developed. In the system, each of the scale-up criteria can be considered as an objective function to be minimized for the single values or the functional relationships along the screw. The scaling is based on process simulation, which is performed using the counter-rotating twin-screw extrusion software TSEM (Twin Screw Extrusion Model). The process is scaled by using GASES_TWIN_ (Genetic Algorithms Screw Extrusion Scaling) software developed for this purpose using genetic algorithms. A Tournament Selection is used as a method of selection. This method offers several advantages over other genetic algorithm selection methods, such as fitness proportionate selection and reward-based selection. These advantages include efficient coding, compatibility with parallel architectures, and adjustable selection pressure.

A scaling up of the extrusion process was carried out to enhance extrusion throughput according to the scaling criteria determined using the single variables of polymer temperature at the die outlet and the relative melting length, and the process variables distribution of the polymer melt temperature and melting. The global objective function had the lowest value for the specified process variables, which means the minimal differences between the variables of the scaled processes and extrusion throughput were significantly increased. The differences between the variables (selected criteria) of the reference process and the target process were small. It can therefore be stated that these processes are similar in terms of these criteria. The temperature and melting profiles were also similar. By scaling up the process, a significant increase in extrusion throughput was attained, which means that the scaling goal was achieved. The solution to the problem of scaling the counter-rotating twin-screw extrusion process presented in this paper is the last, so far unsolved, problem of optimizing and scaling the basic variants of the extrusion process, i.e., flood-fed and starve (metered)-fed single-screw extrusion, as well as co-rotating and counter-rotating twin-screw extrusion.

It can be concluded that the developed scale-up method will work for the problems that are well described by the counter-rotating extrusion simulation program used here. Some examples of experimental verification of this computer program were presented in the literature [81,82,85,86,88,90,91], for example, for polypropylene (PP), low-density polyethylene (LDPE), high-density polyethylene (HDPE), polystyrene (PS), and also for a polyblend of low-density polyethylene and polystyrene (LDPE/PS). However, it is difficult to conclude that the model will work, for example, for reactive extrusion [35,96] or compounding processes [97,98,99,100].

This scaling system will also work for larger scale-up differences. The limitation may be due to the extent to which the simulation program can simulate the process well (predict process outcomes well). As long as the program simulates the process well, the scaling system will work well.

## Figures and Tables

**Table 1:** Dimensions of screws configurations.

**Screws Configuration**							
**Reference**							
**Inside Barrel Diameter, mm**				**Distance between Centres of Screws, mm**			
**34**				**30**			
**Zone No.**	**Zone Length, mm**	**Screw Lead, mm**	**Cylindrical Clearance, mm**		**Clearance, mm**	**Number of Thread Starts**	**Flight Angle, °**
1.	120.0	30.0	0.1		0.5	1	10.0
2.	120.0	20.0	0.1		0.5	1	10.0
3.	120.0	48.0	0.1		0.5	3	10.0
4.	120.0	48.0	0.1		0.5	3	10.0
5.	120.0	48.0	0.1		0.5	3	10.0
6.	30.0	30.0	0.1		0.5	3	10.0
7.	120.0	30.0	0.1		0.5	3	10.0
8.	120.0	30.0	0.1		0.5	3	10.0
9.	120.0	30.0	0.1		0.5	3	10.0
**Target**							
**Inside Barrel Diameter, mm**				**Distance between Centres of Screws, mm**			
**51**				**45**			
**Zone No.**	**Zone Length, mm**	**Screw Lead, mm**	**Cylindrical Clearance, mm**		**Clearance, mm**	**Number of Thread Starts**	**Flight Angle, °**
1.	180.0	45.0	0.1		0.5	1	10.0
2.	180.0	30.0	0.1		0.5	1	10.0
3.	180.0	72.0	0.1		0.5	3	10.0
4.	180.0	72.0	0.1		0.5	3	10.0
5.	180.0	72.0	0.1		0.5	3	10.0
6.	45.0	45.0	0.1		0.5	3	10.0
7.	180.0	45.0	0.1		0.5	3	10.0
8.	180.0	45.0	0.1		0.5	3	10.0
9.	180.0	45.0	0.1		0.5	3	10.0

**Table 2:** Results of scaling up the counter-rotating extrusion.

Scaling-Up Results			
	Extruder		
Single Parameters	Reference	Target	Deviation
Relative melting length	0.242	0.242	0.0%
Polymer melt temperature	207 °C	202 °C	2.4%
Extrusion throughput/ Feeding flow rate	21.88 kg/h	140.0 kg/h	539.9%
Functional parameters (profiles)			
Temperature			
1.	20.00 °C	20.00 °C	0.00%
…	…	…	…
10.	163.26 °C	163.37 °C	0.07%
11.	165.95 °C	166.04 °C	0.05%
…	…	…	…
24.	206.10 °C	199.20 °C	3.35%
25.	207.00 °C	202.02 °C	2.41%
Solid bed, SBP			
1.	1.00	1.00	0.00%
…	…	…	…
5.	0.58	0.54	6.9%
6.	0.48	0.43	10.4%
…	…	…	…
9.	0.08	0.09	12.5%
10.	0.00	0.00	-

## Data Availability

The data presented in this study are available on request from the corresponding author.
